# Macrophage polarization in innate immune responses contributing to pathogenesis of chronic kidney disease

**DOI:** 10.1186/s12882-020-01921-7

**Published:** 2020-07-13

**Authors:** Hewang Lee, Michael B. Fessler, Peng Qu, Jurgen Heymann, Jeffrey B. Kopp

**Affiliations:** 1 Kidney Disease Section, Kidney Diseases Branch, National Institute of Diabetes and Digestive and Kidney Diseases, National Institutes of Health, Bethesda, MD 20892 USA; 2 Institute of Heart and Vessel Diseases, Affiliated Second Hospital of Dalian Medical University, Dalian, 116023 China; 3 Immunity, Inflammation, and Disease Laboratory, National Institute of Environmental Health Sciences, National Institutes of Health, Research Triangle Park, NC 27709 USA

**Keywords:** Apolipoprotein L1, Chronic kidney disease, Immunometabolism, Innate immunity, Macrophage polarization

## Abstract

Chronic kidney disease (CKD) is characterized by inflammation, injury and fibrosis. Dysregulated innate immune responses mediated by macrophages play critical roles in progressive renal injury. The differentiation and polarization of macrophages into pro-inflammatory ‘M1’ and anti-inflammatory ‘M2’ states represent the two extreme maturation programs of macrophages during tissue injury. However, the effects of macrophage polarization on the pathogenesis of CKD are not fully understood. In this review, we discuss the innate immune mechanisms underlying macrophage polarization and the role of macrophage polarization in the initiation, progression, resolution and recurrence of CKD. Macrophage activation and polarization are initiated through recognition of conserved endogenous and exogenous molecular motifs by pattern recognition receptors, chiefly, Toll-like receptors (TLRs), which are located on the cell surface and in endosomes, and NLR inflammasomes, which are positioned in the cytosol. Recent data suggest that genetic variants of the innate immune molecule apolipoprotein L1 (APOL1) that are associated with increased CKD prevalence in people of African descent, mediate an atypical M1 macrophage polarization. Manipulation of macrophage polarization may offer novel strategies to address dysregulated immunometabolism and may provide a complementary approach along with current podocentric treatment for glomerular diseases.

## Background

Chronic kidney disease (CKD) bears a major global health burden, with an estimated prevalence of 8 to 16% of the population worldwide [1, 2]. CKD is manifested by chronic inflammation, with sustained, unsuccessful injury-repair cycles and subsequent fibrosis [3] and these processes involve both protective and pathogenic roles of macrophages [3].

Macrophages are a central component of the innate immune system, which is the first line of defense against endogenous pathogens (defined as pathogens normally present in some tissue compartment) and exogenous pathogens (defined as pathogens not present in a healthy host). Innate responses involve non-specific immune functions (e.g., cytokine release) that are induced upon detection of pathogen-associated molecular patterns (PAMPs [e.g., bacterial lipopolysaccharide]) and host-derived damage-associated molecular patterns (DAMPs [e.g., mitochondrial DNA]) by pattern recognition receptors (PRRs), [4] of which the best studied in association with CKD are membrane-bound Toll-like receptors (TLRs) and cytosolic nucleotide-binding oligomerization domain (NOD)-like receptors (NLRs).

Macrophages may originate from erythro-myeloid progenitors, hematopoietic stem cells or circulating monocytes [5] and display diverse phenotypes in response to the distinct tissue microenvironments in which they reside. M1 and M2 macrophage states have been introduced to describe two extremes of this diversity, which historically have been described as classically activated (M1) and alternatively activated (M2) macrophages, respectively [6]. Experimentally, M1 macrophages are typically induced by exposure to interferon-γ and/or lipopolysaccharide and are considered pro-inflammatory, while M2 macrophages are induced by interleukins (IL) like IL-4, IL-13, and IL-33, and are considered anti-inflammatory [7, 8], which can be further subcategorized into three subgroups: M2a macrophages are induced by IL-4 and/or IL-13, induce anti-inflammatory, wound healing and tissue fibrosis; M2b macrophages are induced by immune complexes in combination of LPS and/or IL-1R ligands, function in immunoregulation; M2c macrophages are induced by IL-10, transforming growth factor (TGF)-β or glucocorticoids, contribute to immunosuppression, matrix deposition and tissue remodeling [7, 8]. As these pure in vitro stimulation conditions are somewhat artificial and reductionist, it is generally recognized that the M1 and M2/M2 subset states are largely idealized and that macrophage polarization in vivo is much more complex, characterized by a continuum of functional phenotypes. Recently, stimulus-specific nomenclature has been proposed for macrophage states, for example, listing the stimulus in brackets such as M [interferon-γ], M [IL-4], or M [IL-Ic], as has specification of macrophage states by cell surface markers, such as CD11b^+^Ly6C^high^, CD14^high^CD16^low^, CD206^−^CD68^+^, and CD206^+^CD68^+^ [9, 10]. Although it is an over-simplification this review will use the ‘M1’ and ‘M2’ dichotomy terminology, in most cases, to align with the existing literature and distinct Th1 and Th2 adaptive immune system of which macrophages is associated.

### Main text

#### Macrophage polarization and chronic kidney diseases

**Fig. 1:**
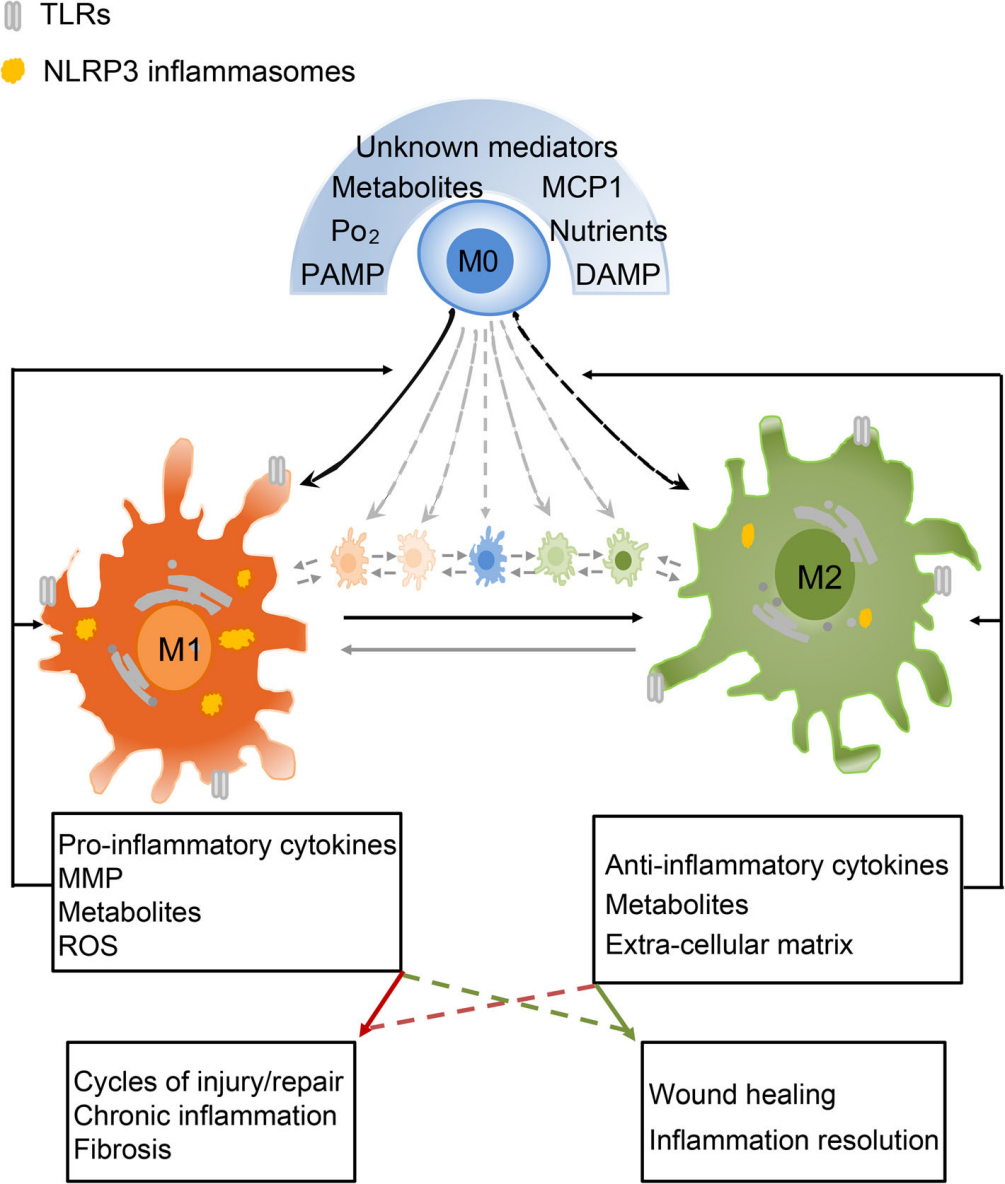
Macrophage polarization in pathogenesis of CKD. Macrophages are activated by DAMPs, PAMPs or other mediators, differentiated and polarized into distinct phenotypes through activation of TLRs, NLRP3 inflammasomes and other receptors. Macrophages demonstrate dramatically diverse phenotypes in inflammation, injury-repair cycles and fibrosis over time, depending on the nature of stimulator(s) in the local environment, injury type, persistence, severity and reparative condition of the kidney. In the early stages of CKD, pro-inflammatory phenotypes could be the major feature of macrophages. If the injury resolves, macrophages switch to an anti-inflammatory phenotype. If the injury cannot be resolved, M1 macrophages remain and M2 macrophages are reduced and may revert to M1 at the site. The products (e.g. cytokines, metabolites etc) released from activated macrophages can join with original stimulator(s) further modulate macrophage function through genetic or epigenetic regulation. DAMP, damage-associated molecular pattern; ECM, extracellular matrix; MCP-1, Monocyte chemoattractant protein-1; PAMP, pathogen-associated molecular pattern; PO_2_, Partial pressure of oxygen; MMP, Matrix metallopeptidase; ROS, reactive oxygen species; TCA cycle, tricarboxylic acid; TLR, Toll-like receptor. Solid line arrow, stimulation; dashed line arrow, not confirmed stimulation

Chronic inflammation plays a leading role in the progression of CKD. Macrophages recognize exogenous PAMPs and/or DAMPs released from damaged tissue and polarize into an M1 phenotype, leading to the release of pro-inflammatory cytokines, chemokines and reactive oxygen species (ROS), and consequent bystander kidney tissue damage [8]. The renal inflammation and injury subsequently initiate healing processes and macrophages repolarize to an M2 phenotype, releasing anti-inflammatory cytokines, chemokines, proangiogenic mediators, and growth factors. If renal inflammation and injury cannot be resolved, resulting in chronic inflammation, [7, 8] the recurring repair and wounding is enhanced [11], with progressive decline of renal function and development of tissue fibrosis (Fig. 1).

M1 macrophages are a characteristic feature of chronic inflammation in CKD. These cells typically display high cell surface expression of CD16, CD32, CD80, CD86, major histocompatibility complex class (MHC) II and IL-1 receptor (IL-1R), production of pro-inflammatory cytokines such as IL-1, IL-6, IL-12, and IL-23, and high expression of oxidative and tissue-remodeling proteins such as inducible nitric oxide synthase (iNOS), matrix metalloproteinase and macrophage-inducible C-type lectin [7]. Plasma pro-inflammatory biomarkers such as TNF were increased in CKD patients in the Chronic Renal Insufficiency Cohort study [12].

However, M2 macrophages are also a major feature of chronic renal inflammation especially during the repair phase, contributing to resolution of inflammation and tissue repair [8]. During the repair phase, M2 macrophages can be switched from M1 macrophages, or originate from in situ proliferation and differentiation from infiltrating monocytes [7, 8, 13]. M2 macrophages function in cell- and tissue- repair and wound healing through antagonizing M1 macrophage function, secreting anti-inflammatory cytokines such as IL-10, IL-22, TGF-β, reducing neutrophil infiltration and suppressing inflammation through Fizz1, Arg1, SOCS1, and SOCS3, and performing clearance of debris and dead cells [8]. A role for M2 macrophages in repair of renal injury has been observed in both in vitro and in vivo experiments; for example, M2 polarization prevents renal injury in the murine model of adriamycin nephropathy [8], and protects the kidney against ischemia-reperfusion injury in Netrin-1 transgenic mice [14].

Evidence from animal models and human CKD patients reveals that M2 rather than M1 macrophages are correlated with progression of fibrosis [7]; however, it is controversial whether M2 macrophages promote or attenuate fibrosis. In the murine unilateral ureteral obstruction (UUO) model, depletion of M2 macrophages reduces renal fibrosis, and adoptive transfer of M2 macrophages promotes accumulation of myofibroblasts expressing smooth muscle α-actin [15] due to secretion of profibrotic factors like TGF-β, galectin-3, and FGF [16]. In the murine anti-glomerular basement membrane model, depletion of M2 macrophages significantly reduced glomerular and interstitial collagen IV deposition, which was accompanied by a reduction in periglomerular smooth muscle α-actin-positive cells [13]. The number of M2 macrophages correlates with the degree of renal fibrosis in IgA nephropathy [17] and diabetic nephropathy [18]. However, in contrast to their promotion of fibrosis, M2 macrophages may promote resolution of renal fibrosis through fibrolytic roles by producing matrix metalloproteinases and degradation of extracellular matrix in the fibrotic kidney [11].

#### Toll-like receptors in macrophage polarization

TLRs are the best-studied macrophage PRRs in CKD. TLR molecules are transmembrane receptors that function either at the cell surface or in intracellular membranes (i.e., endosomes). TLR1–9 are expressed in both humans and mice, while TLR10 is non-functional in mice and TLR11-13 are not expressed in humans [19]. TLR1, 2, 4, 5, 6, and 10 are expressed on the cell surface, and TLR3, 7, 8, 9, 11, 12, 13 are expressed intracellularly [20]. The cellular expression, ligands, signaling and immune response of TLRs are summarized in Table 1 [19–21]. TLRs recognize both PAMPs and DAMPs to shape inflammatory responses and renal injury and fibrotic progression through macrophage polarization in CKD.

**Table 1:** Mammalian Toll-like receptors [19–21]

Toll-like receptor	Expression	Ligand	Signaling	Shaping adaptive immune responses
TLR1^a^	Cell surface monocytes/macrophages dendritic cells B lymphocytes	triacyl lipopeptides lipoproteins lipoarabinomannan	MyD88	Th1 responses
TLR2	Cell surface monocytes/macrophages dendritic cells neutrophils mast cells	glycolipids lipopeptides lipoproteins lipoteichoic acid HSP70 β-glucan zymosan tGPI-mutin	MyD88	Th17 responses Treg responses B cell produce IgM
TLR3	Intracellular compartment dendritic cells B lymphocytes	dsRNA poly I:C	TRIF	MHC I antigen presentation TCR co-receptor CD8 T cell responses
TLR4	Cell surface monocytes/macrophages dendritic cells neutrophils mast cells B lymphocytes	lipopolysaccharides HSPs fibrinogen virus structural protein heparan sulfate hyaluronic acid Mannan nickel opioids glycoinositolphospholipids	MyD88/TIRAP TRIF/TRAM	Th1 responses TNF, IL-6 production B cell IgM production
TLR5	Cell surface monocytes/macrophages dendritic cells	flagellin profilin (controversial)	MyD88	TCR co-receptor Th1, Th17 B cell IgA production
TLR6^a^	Cell surface monocytes/macrophages mast cells B lymphocytes	diacyl lipopeptides lipoprotein LTA PGN zymosan β-glucan	MyD88	Th1 responses
TLR7	Intracellular compartment monocytes/macrophages dendritic cells B lymphocytes	imidazoquinoline compound thiazoquinoline compound AZ12441970 loxoribine bropirimine ssRNA short dsRNA miRNA	MyD88	TCR co-receptor B cell maturation Th17 responses
TLR8	Intracellular compartment monocytes/macrophages dendritic cells mast cells	imidazoquinoline thiazoquinoline loxoribine bropirimine ssRNA(viral) RNA(bacterial) miRNA	MyD88	TCR co-receptor
TLR9	Intracellular compartment monocytes/macrophages dendritic cells B lymphocytes	CpG DNA hemozoin	MyD88	TCR co-receptor B cell maturation Th1 responses
TLR10	Cell surface monocytes/macrophages B lymphocytes	unknown	MyD88	unknown
TLR11	Intracellular compartment monocytes/macrophages	profilin profilin-like molecule	MyD88	Th1 responses IL-12-dependent resistance to T. gondii
TLR12	Intracellular compartment monocytes/macrophages dendritic cells	profilin	MyD88	IL-12-dependent resistance to T. gondii
TLR13	Intracellular compartment monocytes/macrophages dendritic cells	rRNA	MyD88 TAK-1	Antigen cross-presentation

The 13 Toll-like receptors shown here are expressed on the cell surface or within the cell (i.e., in endosomal membranes), by immune cells including monocyte/macrophages, lymphocytes, dendritic cells, and mast cells. Of note, TLR1–10 are expressed in both humans and mice, TLR11–13 are not expressed in humans, while mice express TLR11–13 with TLR10 is non-functional (pseudogene) [19]. These receptors trigger signaling via the pathways shown and the signals shape the response of other immune cells and immune functions as shown. ^a^, indicates the TLR usually functions with formation of heterodimers, i.e., TLR1/TLR2 or TLR2/TLR6

*Abbreviations*: *CD* cluster of differentiation, *HSP* heat shock protein, *IL* interleukin, *MHC* major histocompatibility complex, *MyD88* myeloid differentiation primary response 88, *TAK1* transforming growth factor-Β- activated kinase 1, *TCR* T-cell receptor, *Th* T helper, *TIR* Toll/interleukin-1 receptor, *TNF* tumor necrosis factor, *TRIF* TIR domain-containing adaptor protein including IFN-β, *TRAM* TRIF-related adaptor molecule

Roles of TLRs in inflammation have been observed in both animal models and CKD patients. Activation of macrophage TLR2 induces a pro-inflammatory response and pathogenesis of nephropathy in diabetic mice [22] and inhibition of macrophage TLR2 signaling leads to suppressed diabetic nephropathy [23]. TLR4 expression is significantly higher in stage 3 and 4 CKD patients than healthy controls and is positively correlated with serum levels of TNF-α, IL-6 and MCP-1 in CKD patients [24]. Activation of TLR9 coincides with accumulation of M1 macrophages and increased expression of pro-inflammatory cytokines in the renal interstitial compartment [25]. Of interest, high density lipoprotein from CKD patients activates TLR2 in macrophages, inducing pro-inflammatory cytokines, but is deficient in inducing protective cholesterol efflux [26, 27]. Expression of TLR4 on macrophages and serum IL-6 concentrations are increased during and post-haemodialysis compared to the baseline levels in stage 3 and 4 CKD patients [28]. Taken together, this suggests that activation of TLRs on macrophages not only initiates inflammatory responses and M1 macrophage polarization but also that the uremic environment induces high expression of TLRs, further amplifying pro-inflammatory cytokine production and inflammatory responses in CKD animal models and patients. This increased inflammatory reaction could be one of the major contributors to the high risk of atherosclerosis observed in CKD patients.

The role of TLRs on macrophages in tissue injury is better known in the context of liver wound healing. Deficiency of TLR4 protects against liver injury in various animal models including bile duct ligation and experimental alcoholic and non-alcoholic steatohepatitis; similar findings are also observed in mice deficient in CD14, a TLR4-binding protein, and MyD88 and Trif, TLR4 adaptor molecules, together indicating a critical role for TLR4 in liver injury [29]. A recent study has demonstrated that TLR7 activation also plays important roles in liver injury and progression of early alcoholic liver disease through a Stat3-dependent mechanism [30]. Unlike the liver, the kidney is rarely exposed to bacterial PAMPs. However, levels of endogenous TLR ligands (i.e., DAMPs) increase in the injured kidney. In the kidney, TLR2 and TLR4 are important in the injury of glomerulonephritis, such as lupus nephritis [19]. IL-1 receptor-associated kinase-M, a macrophage-specific TLR inhibitor, improves resolution of kidney injury through reduction of M1 macrophage and TNF-α production [25]. In other contexts, such as bisphosphonate-related osteonecrosis of the jaw, TLR4 inhibition enhances M2 and decreases M1 macrophage polarization, leading to wound healing of the extraction socket [31], and TLR2 activates more strongly in M2 than in M1 macrophages in rheumatoid arthritis patients [32]. TLR4 and other TLRs may thus play roles in the excessive deposition of collagen and other extracellular matrix proteins during the repeated and prolonged injury of kidney tissue in CKD animal models and patients; we posit that this warrants further investigation.

TLRs also contribute to renal fibrosis in chronic renal injury. The crucial pro-fibrotic role of TLR4 has been revealed by TLR4-deficient mice. TLR4-deficient mice exhibit decreased matrix metalloproteinase activity and a significant reduction in fibroblast accumulation and oxidative stress in hypertensive kidneys [33]. Downregulation of TLR4 and its downstream signaling shifts macrophage polarization from an M1 towards an M2 phenotype and ameliorates renal interstitial fibrosis, glomerulosclerosis, and renal functional loss in the early stages of UUO [34] and adriamycin nephropathy in rats [35]. Mutation of TLR4 protects mice from development of inflammation and renal injury including albuminuria, glomerulosclerosis, and renal fibrosis after nephrectomy with angiotensin II infusion, as revealed by C3HeJ TLR4 mutant mice [36]. Deficiency of MyD88, a common adaptor molecule of TLRs, significantly reduces lesions of the glomerular filtration barrier and collagen deposition and leads to reduction of fibrosis after UUO [37].

### NLRP3 inflammasomes in macrophage polarization

Like TLRs, Nod-like receptor (NLR) inflammasomes are PRRs important in the macrophage polarization associated with pathogenesis of CKD [38]. The nucleotide-binding domain, leucine-rich-containing family, pyrin domain-containing-3 (NLRP3) inflammasome is the best characterized member of the NLR inflammasome family. Renal macrophages express all components of NLRP3 inflammasomes, which can sense PAMPs from pathogens or DAMPs released from injured renal tissue including ROS, ATP, extracellular matrix components, oxalate and cholesterol crystals, excess glucose, ceramides, amyloids, urate, and potassium efflux, although in several of these cases, the responses are likely not via direct interaction with NLRP3 [39, 40].

In macrophages, NLRP3 activation can be primed by TLRs (step 1), which activates NF-κB or a non-NF-κB pathway to produce pro-IL-1β and pro-IL-18; step 2 involves the oligomerization of NLRP3 with recruitment of the adaptor molecule apoptosis-associated speck-like protein containing a caspase recruitment domain (ASC) and pro-caspase-1. Active caspase-1 or caspase-11 then cleaves pro-IL-1β and pro-IL-18 to produce mature cytokines IL-1β and IL-18 [41]. Both IL-1β and IL-18 are among the most potent pro-inflammatory cytokines and are important in M1 macrophage polarization [42, 43]. Deficiency of IL-1β attenuates progression of mouse glomerulonephritis with less crescent formation [11], indicating the importance of IL-1β in the pathogenesis of CKD in mice. IL-18, an interferon-γ-inducing factor, is important in lipopolysaccharide-induced macrophage M1 polarization, [44] subsequent inflammation and progression of CKD, [38] and cardiovascular events in CKD patients [45]. NLRP3, IL-1β, and IL-18 are significantly upregulated in chronic kidney disease patients undergoing hemodialysis treatment, indicating that the NLRP3 inflammasome may be activated in and contribute to chronic inflammation in CKD [46].

Chronic inflammation can cause irreversible glomerular and tubular injury and renal functional loss. In 5/6 nephrectomy Munich-Wistar rats, macrophage infiltration is evident after ablation, the NLRP3 inflammasome is activated, and M1 macrophage-related gene expression is increased; furthermore, the glomerulosclerosis index is significantly higher with a progressive increase in albuminuria, creatinine retention, and higher blood pressure compared to the control rats [47]. In nephrocalcinosis-related CKD mice, deposition of oxalate crystal and tubular injury are associated with activation of NLRP3 inflammasomes; inhibition of NLRP3 induces a shift of macrophages from CD45^+^F4/80^+^CD11b^+^CX3CR1^+^CD206^−^, an M1 pro-inflammatory state, to CD45^+^F4/80^+^CD11b^+^CD206^+^TGFβ^−^, an M2 anti-inflammatory phenotype, and attenuates the progression of CKD [48]. In the Tokushima rat model, activation of NLRP3 inflammasomes accelerates macrophage recruitment and M1 polarization, promoting CXCL12 and high mobility group box-1 release in the proximal tubule, and contributing to the progression of diabetic nephropathy [49]. It is also observed clinically that NLRP3 inflammasomes are activated and mature IL-1β is released in anti-neutrophil cytoplasmic antibody-associated glomerulonephritis patients [50].

NLRP3 activation also exacerbates renal fibrosis in CKD. In CKD animal models and human patients, the NLRP3 inflammasome components are upregulated in infiltrating macrophages and other immune cells as well as in podocytes and renal tubular epithelial cells [51]. Inflammasome activation escalates the inflammatory response in macrophages and the crosstalk between macrophages with other immune cells and renal parenchymal cells [52]. In IgA nephropathy mice, NLRP3 inflammasomes in macrophages are activated by IgA immune complexes, leading to the loss of mitochondrial integrity and induction of mitochondrial ROS production [53]. In UUO mice, the severity of renal fibrosis correlates with infiltration of M1 macrophages [54], which is related to the increase of NLRP3 expression and activation [55]. Blockage of NLRP3 attenuates macrophage infiltration, M1 polarization, decrease in gene expression of connexins, TGF-β, *connective tissue growth factor* and *α-smooth muscle actin*, reduction of extracellular matrix deposition, and prevents renal fibrosis and loss of renal function [48, 54, 55].

Activation of intrarenal NLRP3 inflammasomes releases IL-1β, IL-18 and other pro-inflammatory cytokines, which is associated with increased renal inflammation, injury, and fibrosis, and reduced renal function, whereas NLRP3 inhibition induces a shift of infiltrating renal macrophages from a pro-inflammatory and profibrotic phenotype to an anti-inflammatory and anti-fibrotic phenotype, and prevents renal injury and fibrosis in CKD animals and human patients [38, 48]. Therefore, the inflammasome-IL1β/IL-18 axis represents an important mechanism for the pathogenesis of CKD. Of note, recent evidence also demonstrates a role for inflammasome-independent NLRP3 pathways in macrophages and macrophage polarization associated with CKD [40, 48, 54], which needs further investigation.

### Apolipoprotein L1 in macrophage function

**Fig. 2:**
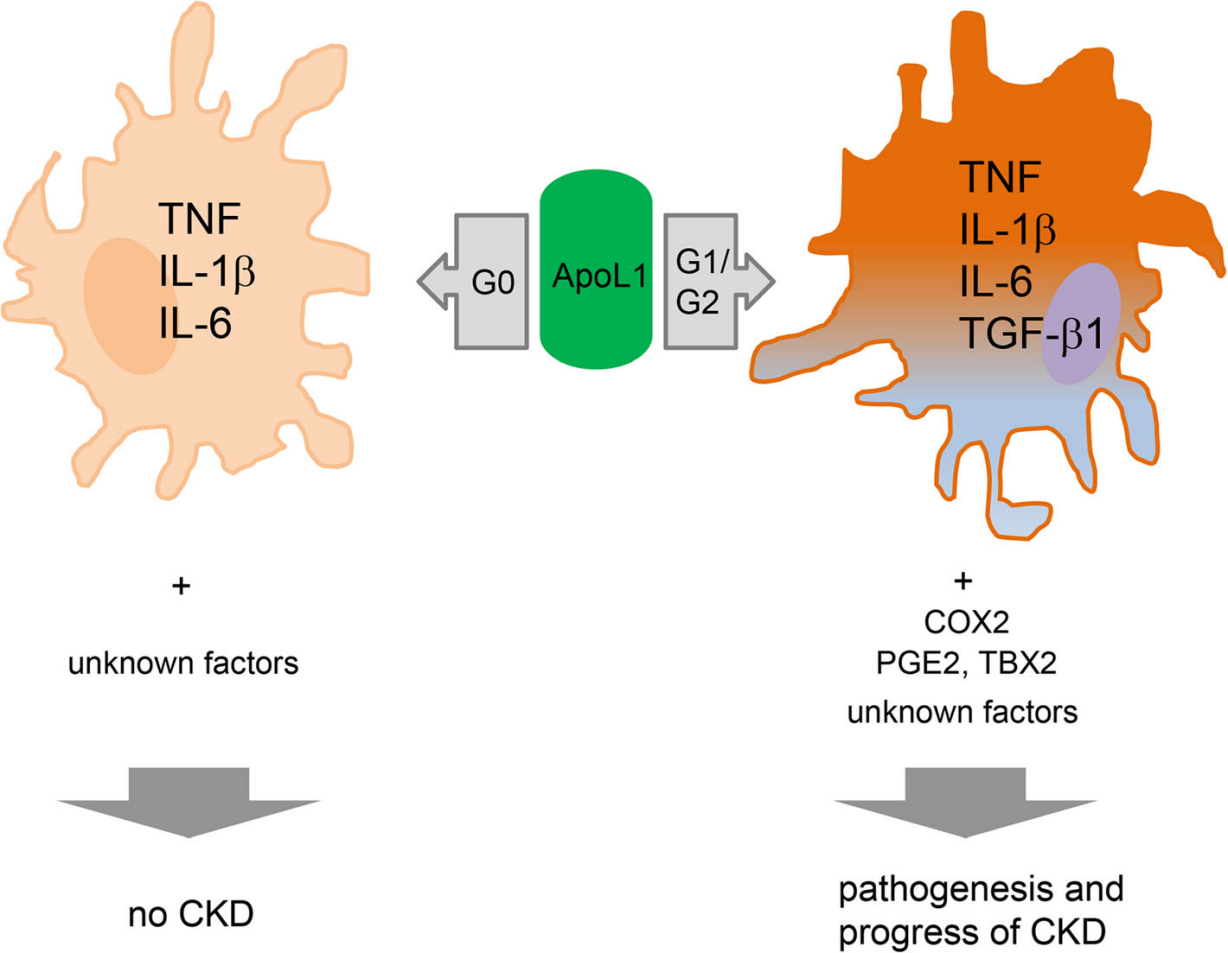
Potential roles of ApoL1 in macrophage polarization. Based on our recent in vitro observation [60], overexpression of all APOL1 variants differentiate macrophages into an atypical M1 state with a marked increase in M1 markers *CD80* (not shown), *TNF*, *IL1β*, and *IL6*. Renal risk variants induce additional TGF-β1 and *CD204* (not shown) or *CD206* (not shown) expression. Renal risk variants also increase PGE2 and TBX2 via the increased expression of COX2, leading to release of TGF-β1. These results demonstrate a role of APOL1 variants in the regulation of macrophage polarization and eicosanoid metabolism, which could promote inflammatory responses and alteration of CKD processing within the injured kidney. Abbreviations: COX2, cyclooxygenase-2; IL, interleukin; PGE2, prostaglandin E2; TBX2, thromboxane B2; TGF, Transforming growth factor; TNF, tumor necrosis factor

Among many other macrophage molecules relevant to innate immunity in the kidney, apolipoprotein L1 (APOL1) is particularly notable for its role in conferring innate immunity to trypanosomal infections and the association of its risk variants with CKD in humans [56]. APOL1 is a minor protein component of human plasma high-density lipoprotein particles and confers innate immunity to trypanosomal infections [57]. APOL1 forms pores in lysosomes [58] and planar lipid bilayers [59] of trypanosoma and this ability to compromise membrane integrity likely contributes to its cellular toxicity in the kidney (Fig. 2). Two common coding variants in *APOL1*, G1 and G2, but not the wild type G0, are associated with increased risk of CKD in individuals with sub-Saharan African ancestry [61, 62]. *APOL* family genes are upregulated by pro-inflammatory cytokines interferon -γ and TNF, and APOL1 can restrict HIV-1 replication in macrophages in vitro [63, 64].

Recent investigation of the molecular mechanisms underlying APOL1-associated CKD suggests increased innate immune function, leading to inflammatory cellular injury or death. *APOL1* high-risk variants increase kidney expression of ubiquitin D and chemokines CXCL9 and CXCL11 [65]. In *APOL1* overexpressing THP-1 macrophages, all three APOL1 isoforms cause monocytes to differentiate into atypical M1 macrophages (Fig. 2) with a marked increase in M1 markers *CD80*, *TNF*, *IL1B*, and *IL6* and a modest increase in the M2 marker *CD163* [60]. It seems this atypical M1 polarization itself is not sufficient to induce CKD, because *APOL1-G0* induces this atypical M1 polarization as well [60]. Over-expression of APOL1 risk variants increases gene expression of the pro-inflammatory cytokines such as TNF, IL-1β and IL-6; increases gene and protein expression of TGF-β; and increases production of prostaglandin E2 [60]. Although *APOL1* renal risk variant-induced pro-inflammatory cytokines are consistent with M1 polarization, there is also an increase in prostaglandin E2 that promotes increased expression of IL-10, mannose-receptor c-type 1 and arginase 2 genes and a decrease in ROS production. This indicates that *APOL1* renal risk variant-mediated expressing macrophages have some M2 features [66].

APOL1 is expressed in macrophages [66, 67] and renal parenchymal cells including podocytes, mesangial cells and endothelial cells [68]. It is not yet clear whether APOL1-associated CKD is caused by direct roles of APOL1 risk variants on podocytes and other renal parenchymal cells or indirectly through innate immune responses by macrophage differentiation and polarization and subsequent interaction with renal parenchymal cells. Recent evidence demonstrates that an antisense oligonucleotide of APOL1 efficiently protects against IFN-γ-induced proteinuria in APOL1-G1 transgenic mice [68], indicating that APOL1-induced M1 macrophage secretion of interferon -γ, M1 polarization and subsequent pro-inflammatory immune responses play at least partial roles in the APOL1-associated CKD. In podocytes, TLR3 activation increases APOL1 expression by upregulating interferon-dependent or interferon-independent pathway [69], and APOL1 risk variants upregulate protein expression of NLRP3 inflammasome components and activation of NLRP3 inflammasomes [70]. Whether APOL1 risk variants interact with TLRs and activate NLRP3 inflammasomes in macrophages and play roles in macrophage polarization and progress of CKD warrants further investigation.

### Immunometabolism in macrophage polarization

**Fig. 3:**
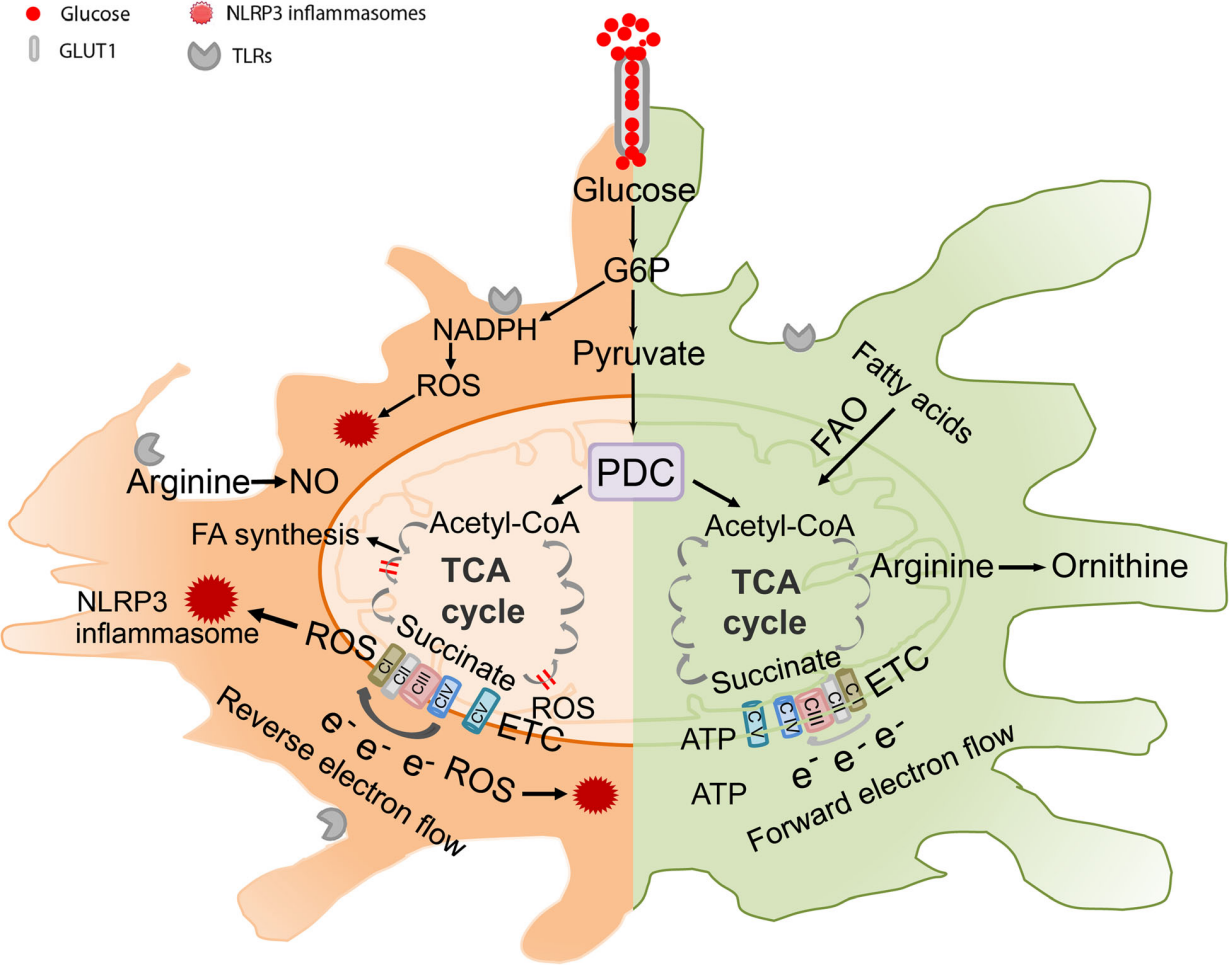
Metabolic reprogramming and macrophage polarization. Consistent with their diverse function and energy demands, macrophages adapt their metabolic programming in response to inflammation, injury, repair and fibrosis in CKD. Glycolysis is increased in activated macrophages with an increase in pyruvate production. In M1 macrophages (left side), most pyruvate is not converted to acetyl-CoA due to the blockage of the TCA cycle; rather, there is flux into the pentose phosphate pathway, generating NADPH, nucleotides and amino acids. Accumulation of citrate drives fatty acid synthesis; the accumulation of succinate leads to induction of HIF-1α, promoting expression of inflammatory and glycolytic genes; reverse electron transfer, along with increased NADPH, and HIF-1α, results in ROS overproduction, and activation of NLRP3 inflammasomes. In M2 macrophages (right side), pyruvate is converted into acetyl-CoA and enters the intact TCA cycle, leading to sustained ATP production via oxidative phosphorylation and up-regulation of genes associated with tissue repair; increased levels of fatty acids enter the TCA cycle leading to an increase in β-oxidation and energy production and a decrease in ROS production due to forward electron transfer. In M2 macrophages, arginase-1 drives the production of polyamines and ornithine in contrast to conversion into citrulline and NO in M1 macrophages. F6P, fructose-6-phosphate; G6P, glucose-6-phosphate; GLUT1, glucose transporter 1; NO, nitric oxide; PDC, pyruvate dehydrogenase complex; PPP, Pentose Phosphate Pathway; FAO, Fatty Acid Oxidation

Cellular metabolism is now recognized to be important in macrophage polarization and function including antigen presentation, clonal expansion, and wound healing [71, 72]. The study of macrophage immunometabolism holds great potential to deepen our understanding of macrophage biology and identify potential therapeutic targets [73, 74]. Different immune activities require that M1 and M2 macrophages adapt their cellular metabolism in order to produce specific metabolites and to meet energy demands (Fig. 3). A key difference between M1 and M2 macrophages is the metabolism of arginine. M1 macrophages metabolize arginine to nitric oxide and citrulline via nitric oxide synthase 2, and these products are pro-inflammatory, cytotoxic, and in turn increase production of reactive oxygen and nitrogen species. In contrast, M2 macrophages express arginases, ornithine decarboxylase and spermidine oxidase, which hydrolyzes arginine and produces ornithine and polyamines [75], suppressing pro-inflammatory responses and promoting repair of tissue damage. In concert with pro-inflammatory nitric oxide production, M1 macrophages upregulate flux through the pentose phosphate pathway and increase production of NADPH, which is required for the generation of NADPH oxidase-derived ROS. On the other hand, M2 macrophages exhibit suppression of the pentose phosphate pathway [76]. The different functions of M1 and M2 macrophages are also associated with characteristic energy metabolic alterations. M1 macrophages mainly rely on glycolysis for energy, while M2 macrophages mainly use mitochondrial oxidative phosphorylation [77].

Stimulation of TLRs induces increased glycolytic metabolism in macrophages, which shows a similar glucose metabolic pattern as classically activated M1 macrophages [78]. This increased glycolysis is thought to allow macrophages to rapidly process carbon from glucose and glutamine to generate biomolecules such as cytokines, chemokines, and other inflammatory mediators during the acute immune response to infection [78]. The metabolic profile can be different with activation of different TLRs in macrophages. For example, activation of TLR4 by LPS increases glycolysis and decreases oxidative phosphorylation, while activation of TLR2 by Pam3CysSK4 increases glycolysis, oxygen consumption rate and mitochondrial activity [79]. NLRP3 inflammasomes are an important regulator of glycolysis [77]. NLRP3 inflammasomes have been shown to sense metabolites such as palmitate, uric acid, and cholesterol crystals and regulate glucose homeostasis [80]. In NLRP3-deficient mice, reduction of pro-inflammatory cytokines is associated with reduction of pro-inflammatory factor MCP-1 and macrophage infiltration, which protected from development of high fat diet-induced obesity and diabetic nephropathy [81]. A recent study reveals that activation of NLRP3 inflammasomes and the subsequent M1 macrophage polarization increases expression of glycolytic enzymes and production of fructose 2, 6-bisphosphate, which can be inhibited by blockage of NLRP3 in macrophages [82]. Whether APOL1 modulates metabolic reprogramming associated with macrophage polarization in CKD is not well understood.

The pyruvate dehydrogenase complex, consisting of mitochondrial enzymes linking glycolysis and the tricarboxylic acid cycle, contributes to the pathogenesis of hypertension in spontaneously hypertensive rats [83], and might play roles in macrophage polarization associated with activation of TLRs, NLRP3 inflammasomes, and expression of APOL1 risk variants. Pyruvate drives the tricarboxylic acid cycle within mitochondria. Accumulation of pyruvate and its conversion into acetyl-CoA support production of mitochondrial ROS via reverse electron transport coupled with oxidative phosphorylation and ATP production, fatty acid synthesis, and lipogenesis, which supports optimal inflammatory responses of M1 macrophages. The expression of enzymes that regulate pyruvate dehydrogenase activity (e.g. pyruvate dehydrogenase kinases and phosphatases) may be concurrently induced in a given M1 or M2 cell type, but are also tightly regulated by the cellular microenvironment (Fig. 3). Identifying novel molecules able to modify the metabolism of polarized macrophages and lymphocytes in the kidney and able to modify the course of progressive CKD represents a promising avenue of investigation.

### Therapeutic approaches

Preclinical and clinical studies have exploited knowledge about macrophage polarization to design and test therapeutic interventions. Innate immune responses through diverse PRRs are critical to macrophage polarization and subsequent signaling cascades and release of pro- and anti-inflammatory cytokines, which shape the progression of CKD. Recent enthusiasm in the effect of innate immune responses on macrophage polarization has led to the targeting of these macrophage polarization checkpoints for potential novel therapy of CKD.

Blockade of TLR receptors and/or of their downstream signaling adaptors has proven an attractive therapeutic strategy for other disorders [84], and thus could also hold promise for CKD. Eritoran, a specific TLR4 inhibitor, has shown promise in attenuation of inflammation of experimental dry eye diseases, influenza infection, and liver ischemia-reperfusion injury [85]. TAK-242, another TLR4 inhibitor, ameliorates progressive tissue fibrosis in preclinical fibrosis animals and in systemic sclerosis patients [86]. OPN-305, a humanized anti-TLR2 antibody, decreased serum IL-6 level in a randomized, double-blind, placebo-controlled clinical trial [87]. Oligonucleotide-based antagonist compounds containing a (5-methyl-dC)p (7-deaza-dG) or (5-methyl-dC) p (arabino-G) motif targeting TLR7, 8, and 9 have been reported to protect renal function in certain CKD in clinical trials [88].

Strategies targeting NLRP3 inflammasomes have mainly focused on the downstream proteins IL-1β and caspase-1. Anakinra, recombinant IL-1Ra, is effective in gout flares in patients with advanced CKD; canakinumab, an anti-IL-1β antibody, results in a significant reduction in the rate of major cardiovascular events in patients with CKD; belnacasan, a selective caspase-1 inhibitor, reduces fibrosis formation in UUO mice; MCC950, a specific NLRP3 inflammasome blocker, has been shown to reduce both IL-1β and IL-18 production and fibrosis in crystal-induced nephropathy mice [89].

Febuxostat and allopurinol, two urate lowering reagents, have been demonstrated to inhibit both TLR and NLRP3 inflammasome activation and subsequent M1 polarization, and are recent promising drugs for CKD or CKD complications. Currently, there are six clinical trials of these urate lowering reagents for CKD patients in the US and UK listed in Clinicaltrial.gov. A pilot trial has so far shown that allopurinol improves renal function in diabetic nephropathy patients, lowers systolic blood pressure, and reduces the progression of renal disease in subjects with CKD [90]. A Phase IV clinical trial to investigate febuxostat, another urate-lowering reagent, on renal function in CKD patients is enrolling participants (ClinicalTrials.gov Identifier: NCT03990363).

The above PRR-targeting agents primarily inhibit inflammation and decrease M1 macrophage polarization. Some agents that directly target macrophages and macrophage polarization, for example, injection of genetically overexpressed IL-4 macrophages [91] or transfusion of IL-4/IL-13-differentiated bone-marrow macrophages [92] have shown a consequent M2 macrophage phenotype and reduction of the degree of renal glomerular inflammation and injury in rodents with nephrotoxic nephritis. Curcumin, a glucose metabolic homeostasis modulator, suppresses the M1 response while promoting an M2 response that enhances macrophage-mediated phagocytosis [93], and could draw attention for potential application in CKD. Other agents investigated target molecules in the local microenvironment such as cytokines and ROS. For example, deletion of TNF from macrophages is associated with lower plasma creatinine and albuminuria in murine diabetic nephropathy; of note, three TNF blockers have been approved for clinical treatment of rheumatic diseases [94]. Tocilizumab, an anti-IL-6 receptor antibody, has been used for treatment of rheumatic diseases, and is now under investigation for the treatment of CKD [95]. Anti-oxidants, which target NF E2-related factor-2/heme oxygenase-1 signaling and contribute to the M1 to M2 phenotype switch, are potential therapeutic targets in diabetic nephropathy [96]. IL-10, TGF-β and IL-4, IL-13-induced M2 macrophages exhibit protection of renal damage in the murine adriamycin nephropathy model [7]; however, the effect of IL-10 is still unresolved in a clinical trial [97]. Anti-fibrotic therapies such as pirfenidone and FG3019 targeting TGF-β and connective tissue growth factor in clinical trials also show intriguing potential for clinical usage [98].

**Fig. 4:**
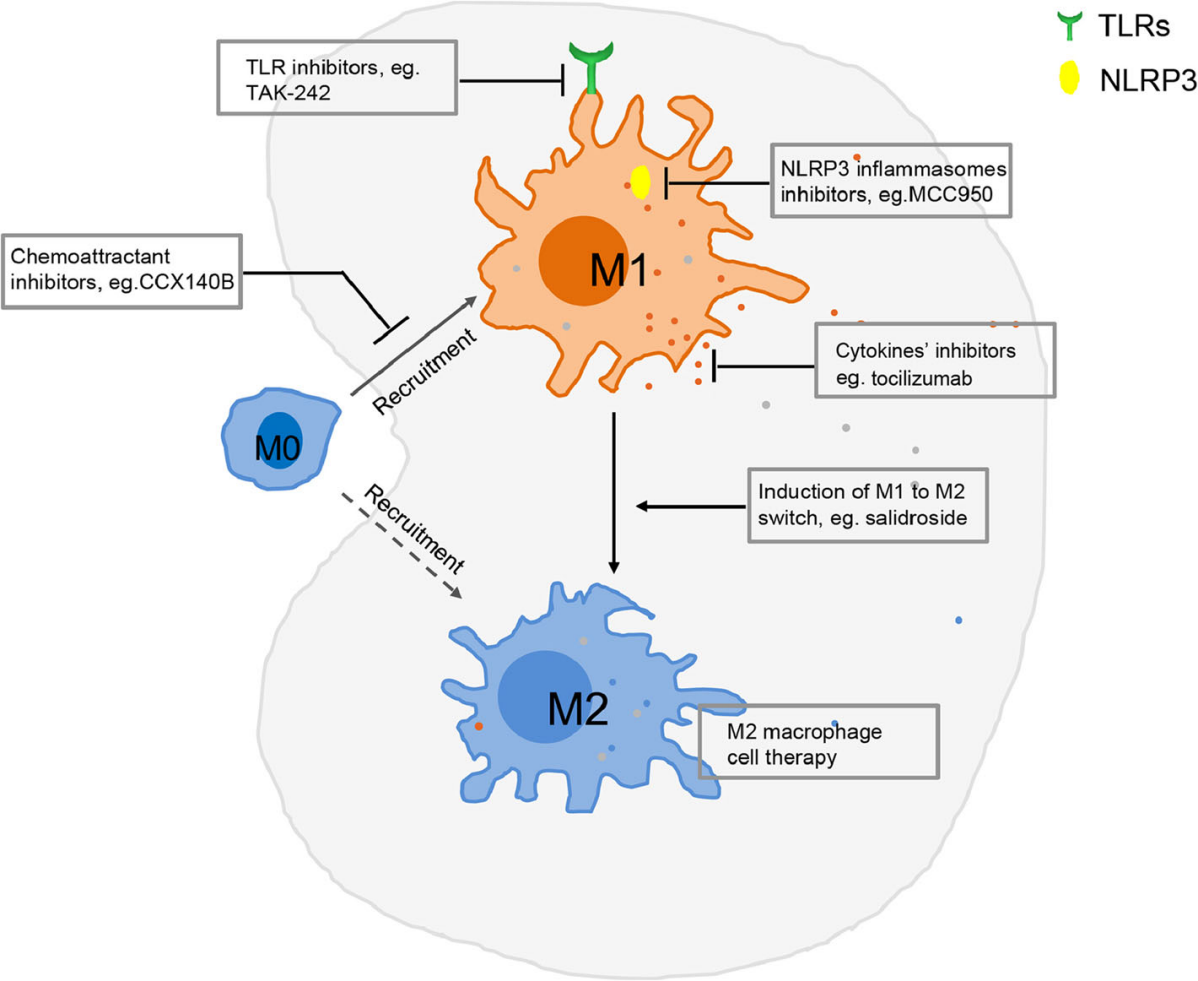
Potential therapeutic targets for macrophage polarization in CKD. Macrophages demonstrate diverse functional phenotypes with two extreme M1 (pro-inflammatory) and M2 (anti-inflammatory) polarization states in response to renal inflammation, injury, repair and fibrosis, which implies that reduction of M1 polarization and induction of M2 polarization could be a promising therapeutic avenue for treatment of CKD. To reduce M1 polarization, inhibitors of TLRs and NLRP3 inflammasomes are promising therapeutic targets in addition to inhibition of macrophage recruitment, proliferation, and transition to myofibroblasts. Some of these agents are currently in Phase II or III trials [7]. To induce M2 polarization, ex vivo IL-4/M-CSF injection, endotoxin precondition in sepsis injured mice, and induction of M1 to M2 switch have been described [8]. However, roles of M2 macrophages in renal anti- versus pro-fibrotic pathology is controversial. Bardoxolone methyl, an activator of the Nrf2 pathway and an inhibitor of the NF-κB pathway, has been evaluated in phase I to III clinical trials for a variety of human diseases including CKD (www.clinicaltrials.gov)

Although most of these agents have not been approved for use in CKD patients, it should be noted that many preclinical and preliminary clinical findings have shown that the modulation of innate immune responses through macrophage polarization is an alternative approach for therapy of CKD (Fig. 4).

## Conclusions

The studies reviewed here demonstrate that the innate immune responses associated with macrophage polarization contribute to the pathogenesis and progression of CKD. Macrophage polarization is highly dynamic and is influenced by innate immune responses, which are altered during CKD progression. TLRs, NLRP3, APOL1 and its risk variants, their downstream signaling, and associated alteration of metabolic processes such as glycolysis and the tricarboxylic acid cycle contribute to the diversity of macrophage polarization and function in CKD. Substantial progress has been made in defining the molecular mechanisms underlying macrophage diversity in vitro. However, limited in vivo studies have been performed to identify macrophage states and to establish their precise roles in the development or amelioration of CKD. Next-generation sequencing-based approaches including whole genome sequencing, whole exome sequencing, and RNA-seq will likely identify various genetic and epigenetic alterations important in macrophage polarization associated with CKD. Exploring the full spectrum of innate and adaptive immune responses and regulation of macrophage and lymphocyte polarization holds tremendous promise to provide novel therapeutic targets for progressive CKD.

## Data Availability

Data for this review were identified by searching MEDLINE, PubMed and references from relevant articles using the search terms “macrophage”, “macrophage polarization”, “M1” or “M2”, “chronic kidney disease” OR “chronic renal disease”, “toll-like receptor”, “NLRP3” and “innate immunity”. To limit the number of references, more recently published papers referring to several previously published articles were cited, if possible. Only articles published in English were selected.

## Abbreviations

APOL1: apolipoprotein L1
ASC: adaptor molecule apoptosis-associated speck-like protein containing a caspase recruitment domain
ATP: adenosine triphosphate
CD: cluster of differentiation
CKD: chronic kidney disease
DAMP: damage associated molecular patterns
IL: interleukin
LPS: lipopolysaccharides
MCP: monocyte chemoattractant protein-1
MHC: major histocompatibility complex
NADPH: nicotinamide adenine dinucleotide phosphate
NLR: cytosolic nucleotide-binding oligomerization domain (NOD)-like receptors
NLRP3: nucleotide-binding domain, leucine-rich-containing family, pyrin domain-containing-3
NOS: nitric oxide synthase
PAMP: pathogen-associated molecular pattern
PRR: pattern recognition receptors
ROS: reactive oxygen species
TGF: transforming growth factor
TLR: toll-like receptors
TNF: tumor necrosis factor
UUO: unilateral ureteral obstruction

## References

[1] Glassock RJ, Warnock DG, Delanaye P (2017). The global burden of chronic kidney disease: estimates, variability and pitfalls. Nat Rev Nephrol..

[2] Kopp JB, Winkler CA (2012). Genetic risk prediction for CKD: a journey of a thousand miles. Am J Kidney Dis..

[3] Engel JE, Chade AR (2019). Macrophage polarization in chronic kidney disease: a balancing act between renal recovery and decline?. Am J Physiol Renal Physiol..

[4] Leemans JC, Kors L, Anders HJ, Florquin S (2014). Pattern recognition receptors and the inflammasome in kidney disease. Nat Rev Nephrol..

[5] Munro DAD, Hughes J (2017). The origins and functions of tissue-resident macrophages in kidney development. Front Physiol..

[6] Murray PJ (2017). Macrophage polarization. Annu Rev Physiol..

[7] Tang PM, Nikolic-Paterson DJ, Lan HY (2019). Macrophages: versatile players in renal inflammation and fibrosis. Nat Rev Nephrol..

[8] Chen T, Cao Q, Wang Y, Harris DCH (2019). M2 macrophages in kidney disease: biology, therapies, and perspectives. Kidney Int..

[9] Murray PJ, Allen JE, Biswas SK (2014). Macrophage activation and polarization: nomenclature and experimental guidelines. Immunity..

[10] Kapellos TS, Bonaguro L, Gemünd I, Reusch N, Saglam A, Hinkley ER, Schultze JL (2019). Human monocyte subsets and phenotypes in major chronic inflammatory diseases. Front Immunol..

[11] Anders HJ, Ryu M (2011). Renal microenvironments and macrophage phenotypes determine progression or resolution of renal inflammation and fibrosis. Kidney Int..

[12] Amdur RL, Feldman HI, Gupta J (2016). Inflammation and progression of CKD: the CRIC Study. Clin J Am Soc Nephrol..

[13] Han Y, Ma FY, Tesch GH, Manthey CL, Nikolic-Paterson DJ (2013). Role of macrophages in the fibrotic phase of rat crescentic glomerulonephritis. Am J Physiol Renal Physiol..

[14] Ranganathan PV, Jayakumar C, Ramesh G (2013). Netrin-1-treated macrophages protect the kidney against ischemia-reperfusion injury and suppress inflammation by inducing M2 polarization. Am J Physiol Renal Physiol..

[15] Feng Y, Ren J, Gui Y (2018). Wnt/β-catenin-promoted macrophage alternative activation contributes to kidney fibrosis. J Am Soc Nephrol..

[16] Meng XM, Mak TS, Lan HY (2019). Macrophages in renal fibrosis. Adv Exp Med Biol..

[17] Ikezumi Y, Suzuki T, Karasawa T (2011). Identification of alternatively activated macrophages in new-onset paediatric and adult immunoglobulin A nephropathy: potential role in mesangial matrix expansion. Histopathology..

[18] Klessens CQF, Zandbergen M, Wolterbeek R (2017). Macrophages in diabetic nephropathy in patients with type 2 diabetes. Nephrol Dial Transplant..

[19] Jimenez-Dalmaroni MJ, Gerswhin ME, Adamopoulos IE (2016). The critical role of toll-like receptors-from microbial recognition to autoimmunity: a comprehensive review. Autoimmun Rev..

[20] Leifer CA, Medvedev AE (2016). Molecular mechanisms of regulation of toll-like receptor signaling. J LeukocL Biol..

[21] Ranoa DR, Kelley SL, Tapping RI (2013). Human lipopolysaccharide-binding protein (LBP) and CD14 independently deliver triacylated lipoproteins to toll-like receptor 1 (TLR1) and TLR2 and enhance formation of the ternary signaling complex. J Biol Chem..

[22] Devaraj S, Tobias P, Kasinath BS, Ramsamooj R, Afify A, Jialal I (2011). Knockout of toll-like receptor-2 attenuates both the proinflammatory state of diabetes and incipient diabetic nephropathy. Arterioscler Thromb Vasc Biol..

[23] Cha JJ, Hyun YY, Lee MH (2013). Renal protective effects of toll-like receptor 4 signaling blockade in type 2 diabetic mice. Endocrinology..

[24] Grabulosa CC, Manfredi SR, Canziani ME (2018). Chronic kidney disease induces inflammation by increasing toll-like receptor-4, cytokine and cathelicidin expression in neutrophils and monocytes. Exp Cell Res..

[25] Lech M, Gröbmayr R, Ryu M (2014). Macrophage phenotype controls long-term AKI outcomes-kidney regeneration versus atrophy. J Am Soc Nephrol..

[26] Kaseda R, Tsuchida Y, Yang HC (2018). Chronic kidney disease alters lipid trafficking and inflammatory responses in macrophages: effects of liver X receptor agonism. BMC Nephrol.

[27] Speer T, Rohrer L, Blyszczuk P (2013). Abnormal high-density lipoprotein induces endothelial dysfunction via activation of toll-like receptor-2. Immunity..

[28] Koc M, Toprak A, Arikan H (2011). Toll-like receptor expression in monocytes in patients with chronic kidney disease and haemodialysis: relation with inflammation. Nephrol Dial Transplant..

[29] Huebener P, Schwabe RF (1832). Regulation of wound healing and organ fibrosis by toll-like receptors. Biochim Biophys Acta..

[30] Stärkel P, Schnabl B, Leclercq S (2019). Deficient IL-6/Stat3 signaling, high TLR7, and type I interferons in early human alcoholic liver disease: a triad for liver damage and fibrosis. Hepatol Commun..

[31] Zhu W, Xu R, Du J (2019). Zoledronic acid promotes TLR-4-mediated M1 macrophage polarization in bisphosphonate-related osteonecrosis of the jaw. FASEB J..

[32] Quero L, Hanser E, Manigold T, Tiaden AN, Kyburz D (2017). TLR2 stimulation impairs anti-inflammatory activity of M2-like macrophages, generating a chimeric M1/M2 phenotype. Arthritis Res Ther..

[33] Pushpakumar S, Ren L, Kundu S, Gamon A, Tyagi SC, Sen U (2017). Toll-like receptor 4 deficiency reduces oxidative stress and macrophage mediated inflammation in hypertensive kidney. Sci Rep..

[34] Chen L, Sha ML, Li D (2017). Relaxin abrogates renal interstitial fibrosis by regulating macrophage polarization via inhibition of toll-like receptor 4 signaling. Oncotarget..

[35] Faustino VD, Arias SCA, Ferreira Ávila V, et al. Simultaneous activation of innate and adaptive immunity participates in the development of renal injury in a model of heavy proteinuria. Biosci Rep. 2018;38:BSR20180762. 10.1042/BSR20180762 PMC6043717 29914975

[36] Souza AC, Tsuji T, Baranova IN, et al. TLR4 mutant mice are protected from renal fibrosis and chronic kidney disease progression. Physiol Rep. 2015;3:e12558. 10.14814/phy2.12558 PMC4600397 26416975

[37] Braga TT, Correa-Costa M, Guise YF (2012). MyD88 signaling pathway is involved in renal fibrosis by favoring a TH2 immune response and activating alternative M2 macrophages. Mol Med..

[38] Hutton HL, Ooi JD, Holdsworth SR, Kitching AR (2016). The NLRP3 inflammasome in kidney disease and autoimmunity. Nephrology..

[39] Chen L, Yao Q, Xu S, Wang H, Qu P (2018). Inhibition of the NLRP3 inflammasome attenuates foam cell formation of THP-1 macrophages by suppressing ox-LDL uptake and promoting cholesterol efflux. Biochem Biophys Res Commun..

[40] Lorenz G, Darisipudi MN, Anders HJ (2014). Canonical and non-canonical effects of the NLRP3 inflammasome in kidney inflammation and fibrosis. Nephrol Dial Transplant..

[41] Yang Y, Wang H, Kouadir M, Song H, Shi F (2019). Recent advances in the mechanisms of NLRP3 inflammasome activation and its inhibitors. Cell Death Dis..

[42] Sieve I, Ricke-Hoch M, Kasten M (2018). A positive feedback loop between IL-1β, LPS and NEU1 may promote atherosclerosis by enhancing a pro-inflammatory state in monocytes and macrophages. Vascul Pharmacol..

[43] Meda Spaccamela V, Valencia RG, Pastukhov O (2019). High Levels of IL-18 and IFN-γ in Chronically Inflamed Tissue in Chronic Granulomatous Disease. Front Immunol..

[44] Novick D, Kim S, Kaplanski G, Dinarello CA (2013). Interleukin-18, more than a Th1 cytokine. Semin Immunol..

[45] Porazko T, Kúzniar J, Kusztal M (2009). IL-18 is involved in vascular injury in end-stage renal disease patients. Nephrol Dial Transplant..

[46] Granata S, Masola V, Zoratti E (2015). NLRP3 inflammasome activation in dialyzed chronic kidney disease patients. PLoS One..

[47] Fanelli C, Arias SCA, Machado FG (2017). Innate and adaptive immunity are progressively activated in parallel with renal injury in the 5/6 renal ablation model. Sci Rep..

[48] Anders HJ, Suarez-Alvarez B, Grigorescu M (2018). The macrophage phenotype and inflammasome component NLRP3 contributes to nephrocalcinosis-related chronic kidney disease independent from IL-1-mediated tissue injury. Kidney Int..

[49] Kim SM, Lee SH, Kim YG (2015). Hyperuricemia-induced NLRP3 activation of macrophages contributes to the progression of diabetic nephropathy. Am J Physiol Renal Physiol..

[50] Tashiro M, Sasatomi Y, Watanabe R (2016). IL-1β promotes tubulointerstitial injury in MPO-ANCA-associated glomerulonephritis. Clin Nephrol..

[51] Chang A, Ko K, Clark MR (2014). The emerging role of the inflammasome in kidney diseases. Curr Opin Nephrol Hypertens..

[52] Andrade-Oliveira V, Foresto-Neto O, Watanabe IKM, Zatz R, Câmara NOS (2019). Inflammation in renal diseases: new and old players. Front Pharmacol..

[53] Tsai YL, Hua KF, Chen A (2017). NLRP3 inflammasome: Pathogenic role and potential therapeutic target for IgA nephropathy. Sci Rep..

[54] Sogawa Y, Nagasu H, Iwase S (2017). Infiltration of M1, but not M2, macrophages is impaired after unilateral ureter obstruction in Nrf2-deficient mice. Sci Rep..

[55] Vilaysane A, Chun J, Seamone ME (2010). The NLRP3 inflammasome promotes renal inflammation and contributes to CKD. J Am Soc Nephrol..

[56] Dummer PD, Limou S, Rosenberg AZ (2015). APOL1 kidney disease risk variants: an evolving landscape. Semin Nephrol..

[57] Vanhollebeke B, Pays E (2010). The trypanolytic factor of human serum: many ways to enter the parasite, a single way to kill. Mol Microbiol..

[58] Perez-Morga D, Vanhollebeke B, Paturiaux-Hanocq F (2005). Apolipoprotein L-I promotes trypanosome lysis by forming pores in lysosomal membranes. Science..

[59] Thomson R, Finkelstein A (2015). Human trypanolytic factor APOL1 forms pH-gated cation-selective channels in planar lipid bilayers: relevance to trypanosome lysis. Proc Natl AcadSci U S A..

[60] Lee H, Roshanravan H, Wang Y, Okamoto K, Ryu J, Shrivastav S, Qu P, Kopp JB (2018). APOL1 renal risk variants induce aberrant THP-1 monocyte differentiation and increase eicosanoid production via enhanced expression of cyclooxygenase 2. Am J Physiol Renal Physiol..

[61] Kopp JB, Nelson GW, Sampath K (2010). APOL1 genetic variants in focal segmental glomerulosclerosis and HIV-associated nephropathy. J Am Soc Nephrol..

[62] Genovese G, Friedman DJ, Ross MD (2010). Association of trypanolytic ApoL1 variants with kidney disease in African Americans. Science..

[63] Taylor HE, Khatua AK, Popik W (2014). The innate immune factor apolipoprotein L1 restricts HIV-1 infection. J Virol..

[64] Kopp JB, Roshanravan H, Okamoto K (2018). Apolipoprotein L1 nephropathies: 2017 in review. CurrOpin Nephrol Hypertens..

[65] Sampson MG, Robertson CC, Martini S (2016). Integrative genomics identifies novel associations with APOL1 risk genotypes in black NEPTUNE subjects. J Am Soc Nephrol..

[66] Montero J, Gomez-Abellan V, Arizcun M, Mulero V, Sepulcre MP (2016). Prostaglandin E2 promotes M2 polarization of macrophages via a cAMP/CREB signaling pathway and deactivates granulocytes in teleost fish. Fish Shellfish Immunol..

[67] Ryu JH, Ge M, Merscher S, et al. APOL1 renal risk variants promote cholesterol accumulation in tissues and cultured macrophages from APOL1 transgenic mice. PLoS One. 2019;14:e0211559. 10.1371/journal.pone.0211559 PMC6472726 30998685

[68] Aghajan M, Booten SL, Althage M, et al. Antisense oligonucleotide treatment ameliorates IFN-γ-induced proteinuria in APOL1-transgenic mice. JCI Insight. 2019;4:e126124. 10.1172/jci.insight.126124 PMC6629101 31217349

[69] Nichols B, Jog P, Lee JH (2015). Innate immunity pathways regulate the nephropathy gene Apolipoprotein L1. Kidney Int..

[70] Jha A, Kumar V, Haque S (2020). Alterations in plasma membrane ion channel structures stimulate NLRP3 inflammasome activation in APOL1 risk milieu. FEBS J..

[71] Buck MD, Sowell RT, Kaech SM, Pearce EL (2017). Metabolic Instruction of Immunity. Cell..

[72] Hobson-Gutierrez SA, Carmona-Fontaine C. The metabolic axis of macrophage and immune cell polarization. Dis Model Mech. 2018;11:dmm034462. 10.1242/dmm.034462 PMC6124558 29991530

[73] Van den Bossche J, Saraber DL (2018). Metabolic regulation of macrophages in tissues. Cell Immunol..

[74] Murray PJ (2020). On macrophage diversity and inflammatory metabolic timers. Nat Rev Immunol..

[75] Rodriguez PC, Ochoa AC, Al-Khami AA (2017). Arginine metabolism in myeloid cells shapes innate and adaptive immunity. Front Immunol..

[76] Nagy C, Haschemi A (2015). Time and demand are two critical dimensions of immunometabolism: the process of macrophage activation and the pentose phosphate pathway. Front Immunology..

[77] Galvan-Pena S, O'Neill LA (2014). Metabolic reprograming in macrophage polarization. Front Immunology..

[78] Rodríguez-Prados JC, Través PG, Cuenca J (2010). Substrate fate in activated macrophages: a comparison between innate, classic, and alternative activation. J Immunol..

[79] Lachmandas E, Boutens L, Ratter JM (2016). Microbial stimulation of different Toll-like receptor signalling pathways induces diverse metabolic programmes in human monocytes. Nat Microbiol..

[80] Hughes MM, O'Neill LAJ (2018). Metabolic regulation of NLRP3. Immunol Rev..

[81] Ahechu P, Zozaya G, Martí P (2018). NLRP3 inflammasome: a possible link between obesity-associated low-grade chronic inflammation and colorectal cancer development. Front Immunol..

[82] Finucane OM, Sugrue J, Rubio-Araiz A, Guillot-Sestier MV, Lynch MA (2019). The NLRP3 inflammasome modulates glycolysis by increasing PFKFB3 in an IL-1β-dependent manner in macrophages. Sci Rep..

[83] Lee H, Abe Y, Lee I (2014). Increased mitochondrial activity in renal proximal tubule cells from young spontaneously hypertensive rats. Kidney Int..

[84] Brown M, O'Reilly S (2018). Innate immunity and Toll-like receptor signaling in the pathogenesis of scleroderma: advances and opportunities for therapy. Curr Opin Rheumato..

[85] Lucas K, Maes M (2013). Role of the toll-like receptor (TLR) radical cycle in chronic inflammation: possible treatments targeting the TLR4 pathway. Mol Neurobiol..

[86] Bhattacharyya S, Wang W, Tamaki Z (2018). Pharmacological Inhibition of toll-like receptor-4 signaling by TAK242 prevents and induces regression of experimental organ fibrosis. Front Immunol..

[87] Reilly M, Miller RM, Thomson MH (2013). Randomized, double-blind, placebo-controlled, dose-escalating phase I, healthy subjects study of intravenous OPN-305, a humanized anti-TLR2 antibody. Clin Pharmacol Ther..

[88] Kandimalla ER, Bhagat L, Wang D (2013). Design, synthesis and biological evaluation of novel antagonist compounds of toll-like receptors 7, 8 and 9. Nucleic Acids Res..

[89] Komada T, Muruve DA (2019). The role of inflammasomes in kidney disease. Nat Rev Nephrol..

[90] Johnson RJ, Nakagawa T, Jalal D, Sánchez-Lozada LG, Kang DH, Ritz E (2013). Uric acid and chronic kidney disease: which is chasing which?. Nephrol Dial Transplant..

[91] Du Q, Tsuboi N, Shi Y (2016). Transfusion of CD206+ M2 macrophages ameliorates antibody-mediated glomerulonephritis in mice. Am J Pathol..

[92] Kluth DC, Ainslie CV, Pearce WP (2001). Macrophages transfected with adenovirus to express IL-4 reduce inflammation in experimental glomerulonephritis. J Immunol..

[93] Mohammadi A, Blesso CN, Barreto GE, Banach M, Majeed M, Sahebkar A (2019). Macrophage plasticity, polarization and function in response to curcumin, a diet-derived polyphenol, as an immunomodulatory agent. J Nutr Biochem..

[94] Kovesdy CP, Kalantar-Zadeh K (2008). Novel targets and new potential: developments in the treatment of inflammation in chronic kidney disease. Expert Opin Investig Drugs..

[95] Choi J, Aubert O, Vo A (2017). Assessment of Tocilizumab (Anti-Interleukin-6 Receptor Monoclonal) as a Potential Treatment for Chronic Antibody-Mediated Rejection and Transplant Glomerulopathy in HLA-Sensitized Renal Allograft Recipients. Am J Transplant..

[96] Landis RC, Quimby KR, Greenidge AR (2018). M1/M2 Macrophages in Diabetic Nephropathy: Nrf2/HO-1 as Therapeutic Targets. Curr Pharm Des..

[97] Ghali JR, Wang YM, Holdsworth SR, Kitching AR (2016). Regulatory T cells in immune-mediated renal disease. Nephrology..

[98] Klinkhammer BM, Goldschmeding R, Floege J, Boor P (2017). Treatment of Renal Fibrosis-Turning Challenges into Opportunities. Adv Chronic Kidney Dis..

